# P-444. Cerebral Small Vessel disease risk in people with HIV and substance use disorder

**DOI:** 10.1093/ofid/ofae631.644

**Published:** 2025-01-29

**Authors:** Folusakin Ayoade, Salma Hernandez, Hansel Tookes

**Affiliations:** University of Miami, Miami, Florida; University of Miami, Miami, Florida; University of Miami Miller School of Medicine, Miami, FL

## Abstract

**Background:**

Cerebral small vessel disease (CSVD) is associated with an increased risk of stroke, dementia, and death. The risk of CSVD in people with HIV (PWH) is twice the general population but the CSVD risk in PWH with substance abuse is poorly defined. With national surveys showing approximately two-thirds of PWH admitting to using an illicit drug in their lifetime, the evaluation of CSVD risk in this population is needful.

**Table 1:**
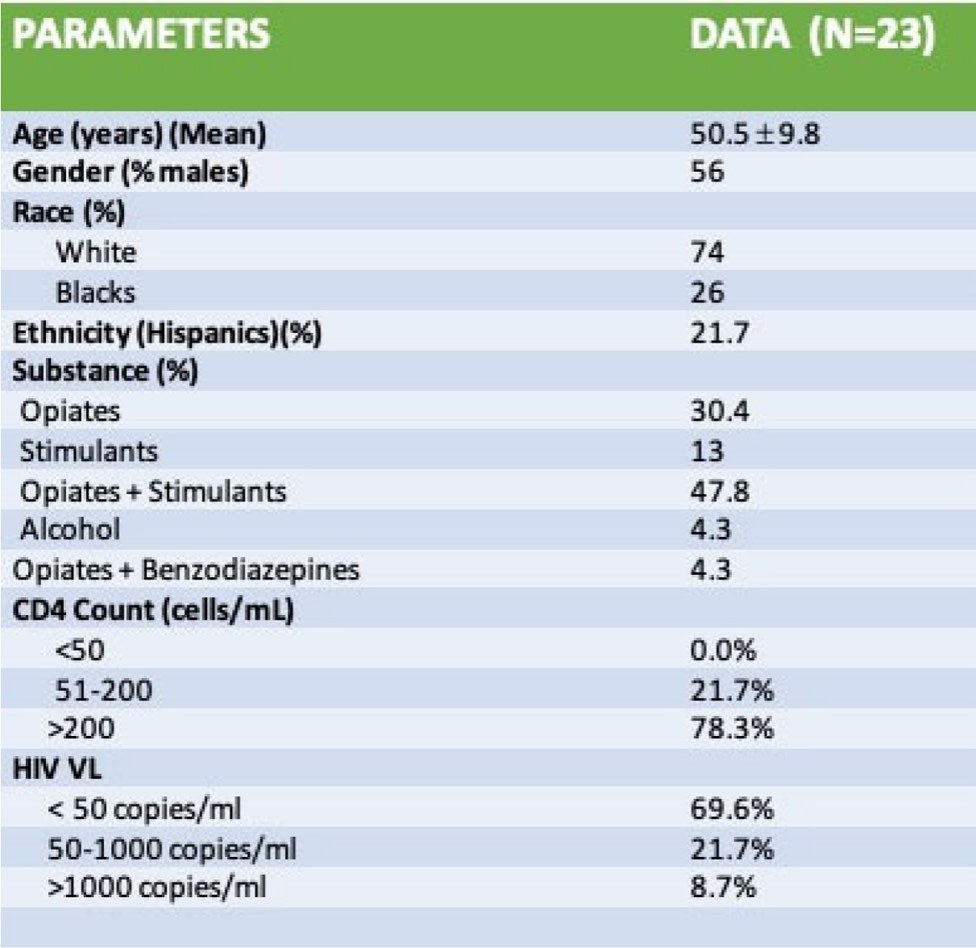
Demographic and Clinical information of CSVD in PWH with substance use disorder (N=23)

**Methods:**

We retrospectively analyzed brain magnetic resonance imaging (MRI) findings of PWH attending our ambulatory HIV needle exchange clinic. Brain MRIs done in the previous 5 years before study analysis were included. CSVD was determined by Cerebral small vessel disease score (0 to 4), assigning 1 point each for the following: (i) presence of lacunar infarct, (ii) microbleeds, (iii) prominent perivascular spaces, and (iv) white matter hyperintensities. The findings suggestive of CSVD such as age-related white matter changes and chronic microvascular ischemia were also included in the CSVD assessment. Substance use disorder (uncontrolled use of a substance despite harmful consequences) was determined to be present if this was included on the patient diagnosis list.

**Table 2:**
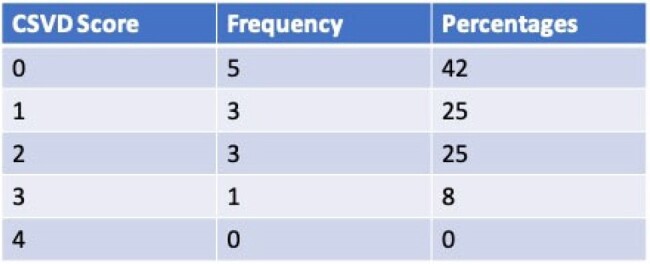
Cerebral Small Vessel disease Score in PWH with substance use disorder. 5 participants had a CSVD score of 0 but "age related white matter disease" on Brain MRI

**Results:**

51 patients (PWH with substance use disorder) were included in the analysis. 23 participants out of this group had brain MRI imaging out of which 12 (52.2%) had CSVD. The mean age was 50 years. The demographic and clinical data of the cohort are shown in Table 1. The CSVD score ranged from 0 to 3 with an average score of 1. Table 2 illustrates the CSVD score of the cohort. 5 participants had a CSVD score of 0 but a brain MRI report of “age-related white matter changes”. The Pearson Correlation between age and CSVD score was 0.057 with a p-value of 0.86.

**Conclusion:**

52% of PWH who use substance have CSVD on MRI brain imaging. This could translate to an increased risk of stroke, cognitive impairment, and death if not recognized early and aggressively addressed. CSVD score provides objective quantification of CSVD but age-related white matter changes, which are not captured on this score may be equally important especially as PWH continues to age. A systematic approach for screening for CSVD in PWH with substance use disorder is warranted to minimize the unfavorable outcomes associated with this cohort.

**Disclosures:**

**All Authors**: No reported disclosures

